# Agreement among physiotherapists in assessing patient performance of exercises for low-back pain

**DOI:** 10.1186/s12891-018-2173-9

**Published:** 2018-07-27

**Authors:** Aurore Hermet, Alexandra Roren, Marie-Martine Lefevre-Colau, Adrien Gautier, Jonathan Linieres, Serge Poiraudeau, Clémence Palazzo

**Affiliations:** 1 Service de rééducation et réadaptation de l’appareil locomoteur et des pathologies du rachis, Hôpital Cochin AP-HP, Université Paris Descartes, PRES Sorbonne Paris Cité, Paris, France; 2 CRESS, UMR 1153, INSERM, Paris, Institut fédératif de recherche handicap, INSERM/CNRS, Paris, France; 3 Institut Fédératif de Recherche Handicap, INSERM/CNRS, Paris, France

**Keywords:** Physical therapists, LowBack pain, Rehabilitation, Exercise therapy, Agreement

## Abstract

**Background:**

There is no agreement for the performance assessment of patients who practice exercises.. (2 points to withdraw) This assessment is currently left to the physiotherapist’s personal judgement. We studied the agreement among physiotherapists in rating patient performance during exercises recommended for chronic low-back pain (LBP).

**Methods:**

A vignette-based method was used. We first identified ten exercises recommended for LBP in the literature. Then, 42 patients with chronic LBP participating in a rehabilitation program were videotaped during their performance of one of the ten exercises. A vignette was an exercise video preceded by clinical information. Ten physiotherapists from primary (4) and tertiary care (6) viewed the 42 vignettes twice, one month apart, and rated patient performance from zero (worse performance) to ten (excellent performance) by considering the position and duration of the contraction or stretching. Intra-class correlation coefficients (ICCs) and 95% confidence intervals (95% CIs) were computed to assess inter- and intra-rater reliability.

**Results:**

The overall inter-rater agreement was fair (ICC 0.48 [95% CI 0.33–0.56]) but was better for stretching exercises (0.55 [0.35–0.64]) than strengthening exercises (0.42 [0.20–0.52]) and for tertiary-care physiotherapists (0.66 [0.54–0.76]) than primary-care physiotherapists (0.28 [0.09–0.37]). The intra-rater agreement was overall good (0.72 [0.57–0.81] to 0.88 [0.79–0.94])*.* It was better for stretching exercises (from 0.68 [0.46–0.81] to 0.96 [0.91–0.98]) than strengthening exercises (from 0.68 [0.38–0.84]) to 0.82 [0.56–0.92]).

**Conclusion:**

The agreement in rating patient performance of exercises for LBP is good among physiotherapists trained in managing LBP but is low among non-trained physiotherapists.

**Electronic supplementary material:**

The online version of this article (10.1186/s12891-018-2173-9) contains supplementary material, which is available to authorized users.

## Background

Exercise therapy decreases pain and improves function in musculoskeletal diseases [1–3]. Individually designed exercise programs are effective in healthcare settings [1, 4]. The exercise program is usually learned during supervised physical therapy sessions and is performed at home by the patient alone, so the patient must be able to self-actualize the exercises at the end of supervised sessions.

There is no standardised way to assess patient performance during exercises. In practice, the assessment is left to the physiotherapists’ personal judgement. This judgement may result from an unconscious integration of various data such as their own beliefs and experience, patient characteristics (age, comorbidities), exercise characteristics, and the relationship with the patient [5]. Better assessment of patient performance could help to improve the teaching of exercises and determine how many physiotherapy sessions are required for one patient, to propose a more personalized treatment. Indeed, if the number of supervised sessions is not sufficient, the treatment can be ineffective and patients can stop home exercises because they do not feel able to practice alone. In contrast, if the number of supervised sessions is greater than needed, the exercises will be a waste of time both for the physiotherapist and the patient.. ( 2 points to withdraw) As well, we need to better understand why treatment fails for some patients and whether home exercises are correctly performed to better adapt the treatment strategy: if exercises are correctly performed, other treatments may be considered; otherwise, new learning sessions and advice may be necessary. Finally, patients may have doubts about their performance and should be advised promptly to avoid stopping the exercises. This advice could improve adherence to home exercises, which is a common problem in musculoskeletal-disease rehabilitation [6–8].

Low-back pain (LBP) is highly prevalent [9], disabling, and costly [10, 11] and represents the first cause for needing a physiotherapist in France [12, 13]. Numerous studies have shown the effectiveness of exercise therapy in reducing pain and improving function with this condition [1, 14, 15]. Therefore, LBP is an ideal condition to evaluate whether physiotherapists’ judgement can be trusted and is reproducible.

The aim of this study was to assess the agreement among physiotherapists in rating patient performance during exercises recommended for LBP.

## Methods

This is an intra- and inter-reliability study. Case-vignettes were used to study physiotherapists agreement [16] because these allow for different health providers to assess the same exercise performed by the same patient.

### Development of vignettes

#### Identification of exercises to translate into vignettes

We identified the exercises recommended in LBP by a non-exhaustive literature review. One author (CP) searched MEDLINE and PEDRO databases for articles evaluating the effectiveness of exercises in LBP that were published in English from 1982 to 2012.

**Fig. 1:**
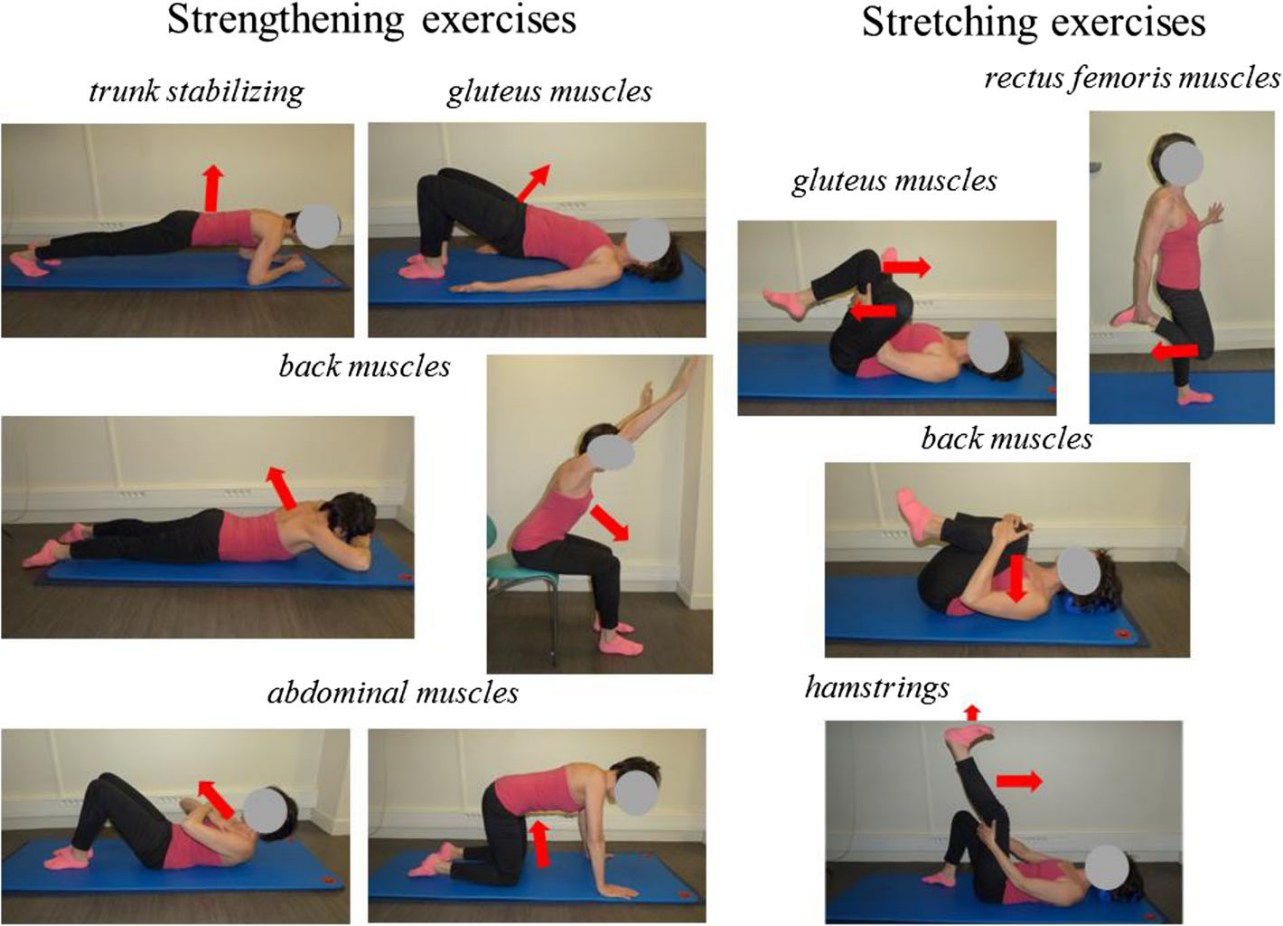
Exercises for strengthening and stretching

From the articles obtained, the steering committee of the study (including one physical medicine and rehabilitation physician, one rheumatologist and one physiotherapist expert in LBP) selected ten exercises: six strengthening exercises (two for back muscles, two abdominal muscles, one gluteus muscles and one trunk stabilizing exercise) with alternating contraction/rest periods of five seconds (five repetitions) and four stretching exercises (one for hamstrings, one gluteus muscles, one back muscles and one rectus femoris muscles) with a stretch of at least 20 s (Fig. 1).

#### Creation of vignettes

A vignette included brief clinical information for the patient (age, main co-morbidities that could affect the achievement of the exercise [e.g., knee osteoarthritis can affect the ability to kneel]), a short description of the exercise (e.g., used for strengthening back muscles), and a video of a patient performing the exercise.

After giving their consent, 42 patients with nonspecific chronic LBP participating in a supervised rehabilitation program in a tertiary-care hospital (Cochin hospital, Paris, France) were videotaped while they performed one of the specific exercises they had learned (at least four different patients performed the same exercise). An example of a vignette is shown in Additional file 1. The acquisition of videos was highly standardised to ensure reproducibility (Additional file 2).


Additional file 1: An example of a vignette with a short video of a patient performing the exercise. (MP4 119063 kb)


### Participants

All physiotherapists working in the rehabilitation department of the tertiary-care Cochin hospital were informed of the study and were asked to participate on a voluntary basis [15]. Six physiotherapists accepted to participate.

Six physiotherapists (staff personal contacts) working in primary care centres were informed of the study by e.mails. Four accepted to participate.

The experience was self-reported. They were asked: “Among your patients, what percentage of them have low back pain?” We considered physiotherapists to be experienced when more than 50% of their patients had low back pain and low experienced when less than 50% of their patients had low back pain.

### Study design

**Fig. 2:**
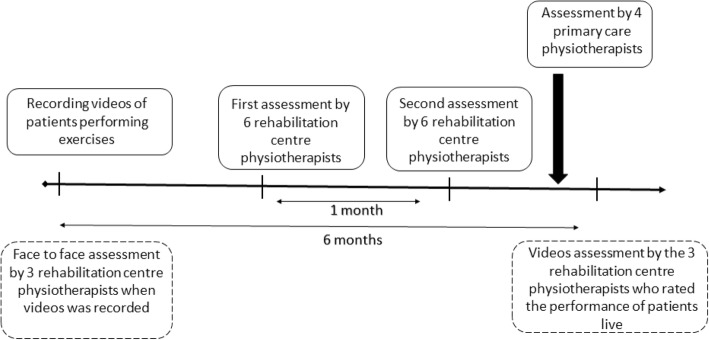
Diagram of the study design

The Fig. 2 shows the study design. Before scoring the vignettes, the physiotherapists provided the following information: age, gender, time working in the current job, experience in the management of LBP (proportion of patients with LBP they daily managed).

All rehabilitation center physiotherapists scored the 42 vignettes twice. The first scoring determined the inter-rater agreement and the second scoring (one month apart) determined the intra-rater agreement. For each vignette, they received the following instruction: “We ask you to assess the patient’s ability to perform an exercise recommended for LBP. Taking into account the patient’s position during the whole exercise, the duration of the contraction or the stretching, could you please note from zero (worse performance) to ten (excellent performance) the patient’s ability to perform the following exercise?” The vignettes were saved on a personal computer and could be viewed individually by participants for one week. Participants were asked not to talk about the topic during the study period. The answers were anonymous. To assess intra-rater agreement, participants were asked one month later to rate the same vignettes in another random order and blind of their first answers.

The primary care physiotherapists received the same 42 vignettes by e-mail and rated them once. Because this process was time-consuming, a second scoring was not possible. Only inter-rater agreement could be assessed.

Finally, three other rehabilitation centre physiotherapists were asked to rate the performance of 16 patients doing exercises while they were being videotaped. Six months later, they rated the exercise videos of the same patients so we could compare the face-to-face assessment with the scoring of the exercise video*.*

### Statistical analysis

We estimated the number of vignettes and participants needed for physiotherapists to assess agreement on the precision of the intra-class correlation coefficient (ICC), and on feasibility considerations (time needed to build a vignette, time needed for scoring, number of participants available). With 42 vignettes, each scored twice, and an expected inter-observer ICC of 0.60, the expected 95% confidence interval (95% CI) would be about 0.4 [17].

Data are described as median (range). As data had a near normal distribution, ICCs were used to assess intra- and inter-rater agreement. ICC estimates were calculated based on a single measurement, consistency, two-way mixed-effects model (ICC (3,1)). The bootstrap procedure (bias-corrected and accelerated bootstrap) was used to estimate 95% CIs. An ICC of 0 indicates chance agreement and 1 perfect agreement. We defined agreement as poor, ICC < 0.4; fair, 0.4 to 0.59; good, 0.6 to 0.74; and excellent, ≥0.75 [18]. Bland and Altman plotting was used to assess the quality of concordance among physiotherapists by the amplitude of the agreement intervals, with the upper and lower limits of agreement defined as the mean difference plus and minus 1.96 times the standard deviation of the differences.

Analyses involved use of R v3.1.2 (statistical software). Written consent was obtained for all participants. The study was approved by the local ethics committee (Comité d’évaluation éthique de l’INSERM (IRB00003888)).

## Results

Ten physiotherapists participated in the study: six from the rehabilitation department of Cochin hospital and four from primary care. The median age was 26 years (range 23–42) and six were women. The physiotherapists from primary care were less experienced in managing LBP than those from tertiary care. Patients with LBP represented 80% of the patients managed in Cochin hospital, while in primary care patients with LBP were not always majority. Only one in four primary care physiotherapists had 60% of his patients with LBP. *(*Table 1*).*

**Table 1:** Physiotherapist (PT) characteristics

	*All PTs* *n = 10*	*Rehabilitation centre PTs* *n = 6*	*Primary-care PTs* *n = 4*
Age (years), median (range)	26 (23–42)	26 (23–42)	26 (25–29)
Female, n (%)	6 (60)	4 (67)	2 (50)
Time working in the current job (years), median (range)		6 (3–18)	4 (2–7)
Personal experience in LBP management: None Low (< 50%) High (> 50%)		high experience	low experience

### Inter-rater agreement

Overall, the inter-rater agreement was fair for the ten physiotherapists (ICC 0.48 [95% CI 0.33–0.56]) *(*Table 2*).* The agreement was better for stretching exercises (0.55 [0.35–0.64]) than strengthening exercises (0.42 [0.20–0.52]), with an overlap of CI.

**Table 2:** Inter-rater agreement for PTs rating patients’ ability to perform exercises

*Agreement*	*All PTs*	*Rehabilitation centre PTs*		*Primary-care PTs*
		First assessment	Second assessment*	
Global	0.48 (0.32–0.56)	0.66 (0.54–0.76)	0.70 (0.58–0.77)	0.28 (0.09–0.37)
For strengthening exercises	0.42 (0.2–0.52)	0.58 (0.32–0.71)	0.65 (0.43–0.77)	0.34 (0.07–0.48)
For stretching exercises	0.55 (0.35–0.64)	0.73 (0.56–0.82)	0.73 (0.57–0.82)	0.21(− 0.01–0.28)

*Data are intraclass correlation coefficients (ICCs) and 95% confidence intervals (95% CIs)*

*1 month later

The agreement among physiotherapists from tertiary care was good (ICC 0.66 [0.54–0.76]) *(*Table 2*).* It was better for stretching exercises (0.73 [0.56–0.82]) than strengthening exercises (0.58 [0.32–0.71]) with an overlap of CI. During the second scoring of the vignettes (one month later), the agreement increased to 0.70 [0.58–0.77]); the agreement for strengthening exercises improved (0.65 [0.43–0.77]) but remained stable for stretching exercises (0.73 [0.57–0.82]) with an overlap of CI.

By contrast, the inter-rater agreement among primary care physiotherapists was poor (ICC 0.28 [0.09–0.37]) *(*Table 2). The agreement was better for strengthening exercises (0.34 [0.07–0.48]) than stretching exercises (0.21 [− 0.01–0.28]) but remained low with an overlap of CI. One primary-care physiotherapist scored the vignettes differently from the others (higher or lower scores), especially for stretching exercises. Without this outlier, the global agreement was better (0.46 [0.23–0.51]) but still low; the agreement for strengthening exercises was low (0.29 [95% CI 0–0.46]) but was good for stretching exercises (0.70 [0.31–0.66]).

**Fig. 3:**
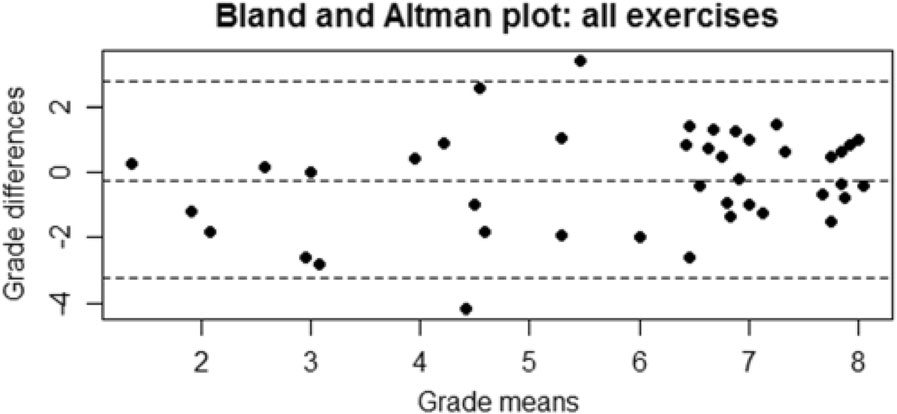
Bland and Altman plot for agreement among all physiotherapists (*n* = 10) Horizontal dotted line is the mean difference, and upper and lower lines are 95% confidence intervals for limits of agreement.

The amplitudes of the agreement intervals on a Bland– Altman plot (Fig. 3) indicated the

quality of concordance among physiotherapists. The 5 and 95% CIs correspond to the limits of agreement. For the global preference, the graphs indicated that the mean of the differences among the raters was very close to zero, with plots having a double funnel shape. It shows a greater concordance for the highest scores (the most successful exercises) (*Bland and Altman plots for strengthening and stretching exercises in* Additional file 3).

### Intra-rater agreement

The intra-rater agreement was very good for all physiotherapists (ICC 0.72 [95% CI 0.57–0.81] to 0.88 [0.79–0.94]) *(*Table 3*).* It was better for stretching exercises (from 0.68 [0.46–0.81] to 0.96 [0.91–0.98]) than strengthening exercises (from 0.68 [0.38–0.84] to 0.82 [0.56–0.92]).

**Table 3:** Intra-rater agreement for PTs rating patients’ ability to perform exercises

*PT number*	*Global*	*For strengthening exercises*	*For stretching exercises*
1	0.74 (0.58–0.84)	0.68 (0.38–0.84)	0.78 (0.57–0.91)
2	0.72 (0.57–0.81)	0.71 (0.43–0.84)	0.68 (0.46–0.81)
3	0.86 (0.76–0.92)	0.79 (0.57–0.91)	0.92 (0.86–0.95)
4	0.82 (0.73–0.89)	0.81 (0.71–0.87)	0.84 (0.67–0.96)
5	0.86 (0.73–0.93)	0.82 (0.48–0.94)	0.88 (0.73–0.94)
6	0.88 (0.79–0.94)	0.82 (0.56–0.92)	0.96 (0.91–0.98)

*Data are ICCs and 95% CIs*

### Comparison between video and face-to-face assessment

The three physiotherapists who rated the performance of patients live and on video all work in the rehabilitation department of Cochin hospital. They did not participate in the rest of the study. They were older (median 31 years [range 29–45]) and more experienced (median time working in the current job seven years [range 6–20]) than the other physiotherapists.

The intra-rater agreement was excellent and very good for two physiotherapists (ICC 0.93 [95% CI 0.38–1.00] and 0.71 [0.1–0.9]), whereas the third one had low agreement (0.39 [− 0.34–0.72]) (Additional file 4).

## Discussion

This study shows a good agreement among tertiary care physiotherapists, experienced in the management of LBP, but a low agreement among primary care physiotherapists, who were less experienced in the management of LBP. The reliability was greater during the second assessment, suggesting that training physiotherapists can improve their agreement in assessing patient performance of exercises for low-back pain.

These discrepancies may arise from a recruitment bias, as the physiotherapits of the rehabilitation centre have the same background and are used to manage a very specific population of patients (ie those who are not improved after a primary care physiotherapy), whereas the primary care physiotherapists may have different background and expectations. As well, we found better agreement for stretching than strengthening exercises, which suggests that stretching exercises are easier to score than strengthening exercises, which may require more feedback.

Although the performance of patients during therapeutic exercises may be a strong predictor of the effectiveness of exercises, it has never been studied previously. When the patient is learning the personalised exercise program during supervised sessions, the ability to perform the exercises should be assessed regularly. Our study suggests that this assessment could be easily performed by physiotherapists experienced in LBP management or with specific training. The adequate number of supervised sessions could be adapted to each patient, for more personalized care, which may be more effective. Moreover, regularly assessing patient performance when they practice therapeutic exercises at home could help determine when exercises are no longer performed adequately (“unlearning” curve) and when refreshing supervised sessions are required.

Adherence is a main issue for exercise therapy programs, especially home-based programs [19]. The World Health Organization has defined adherence as “the extent to which a person’s behaviour taking medication, following a diet, and/or executing lifestyle changes, corresponds with agreed recommendations from a health care provider” [20]. By extension, exercise adherence is often considered the extent to which a patient acts in accordance with the advised interval, exercise dose, and exercise dosing [21]. This definition does not take into account the performance of the patient when performing the prescribed exercises, which may explain why adherence is almost never reported in studies assessing the effectiveness of home-based exercise programs. For example, in a systematic review of interventions to improve adherence to exercise for chronic musculoskeletal pain, only 4 of 42 studies used the accuracy of exercises performed to rate adherence. However, an accurate reporting of adherence seems essential to better address the treatment efficacy of home-based exercises programs in clinical studies [22]. Consequently, future studies evaluating the effectiveness of therapeutic exercises should include an assessment of patient performance. Patient performance could be assessed by physiotherapists on a numeric rating scale from zero to ten.

We also wondered if these results could be transposed to a face-to-face assessment, without a video. The intra-rater agreement was high for two physiotherapists but low for the third one, perhaps because he was aphysiotherapist manager and therefore less involved in patient care and did not directly participate in teaching exercises to patients (Additional file 4). Thus, the judgment of the videos was close to live assessment. This finding has two major advantages: first, our results could be transposed to a face-to-face assessment and second, patient performance could be assessed via “telemedicine”, so that patients could be advised by a physiotherapist from their home.

This work has some limitations. A key limitation of this study as that COnsensus-based Standards for the selection of health (COSMIN) was not used to inform study design and decisions’. The number of physiotherapists was small, and there was a recruitment bias for the rehabilitation centre physiotherapists as they worked together. That is why we also wanted to include primary care physiotherapists. The confidence intervals could be wide because the numbers of physiotherapists were small. When the confidence intervals overlap, it was not possible to conclude that there is a definite difference But there was no overlap of confidence intervals when comparing the ICCs of the tertiary care physiotherapists with the ICCs of the primary care physiotherapists, suggesting a significative difference of reliability between them.. Finally, we focused on the exercises recommended for one particular condition, LBP, because this is a common problem with a significant socioeconomic impact. These results should be confirmed for other disorders, such as knee osteoarthritis or rotator cuff diseases.

## Conclusion

The agreement among physiotherapists experienced in managing musculoskeletal disorder is good for using a ten-point scale to rate patient performance during exercises recommended for LBP. Training of less experienced physiotherapists is necessary. A ten-point scale could be used to assess patients performance in clinical studies evaluating the effectiveness of exercise therapy in LBP, but also in real life to determine the adequate number of physiotherapy sessions required and to help better understand the unlearning phenomenon of exercises.

This study is providing initial insights to determine the agreement among physiotherapists in assessing patient performance. Future studies will be needed to evaluate these findings in another population of physiotherapists and in other musculoskeletal disorders.

## Additional files

Additional file 2: How videos were obtained. Videos were obtained in the same room with the same placement. During the editing, there were no corrections. We blurred faces to preserve patient anonymity. (PDF 114 kb)

Additional file 3: Bland and Altman plots of agreement for all physiotherapists for strengthening and stretching exercises. (PDF 163 kb)

Additional file 4: Intra-rater agreement among three rehabilitation centre physiotherapists (face-to-face assessment vs video assessment). (PDF 106 kb)

## Abbreviations

95% CI: 95% confidence interval
ICC: Intra-class correlation coefficient
LBP: Low-back pain
