# Supplementary material for: Agreement among physiotherapists in assessing patient performance of exercises for low-back pain
Source: BMC Musculoskelet Disord. 2018 Jul 27;19:265. doi: 10.1186/s12891-018-2173-9 (PMC6064172; doi:10.1186/s12891-018-2173-9)

Appendix 2: How videos were obtained.

Videos were obtained in the same room with the same placement. During the editing, there were no corrections. We blurred faces to preserve patient anonymity.

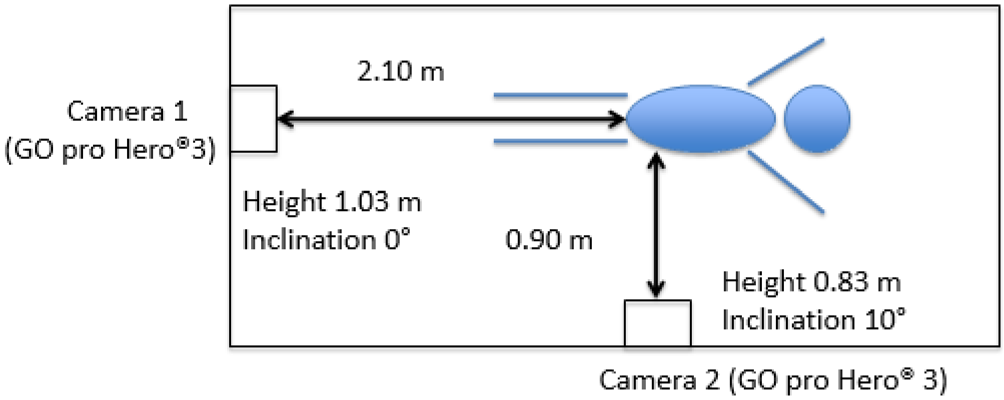

Supplement: Supplementary file 2 — How videos were obtained. Videos were obtained in the same room with the same placement. During the editing, there were no corrections. We blurred faces to preserve patient anonymity. (PDF 114 kb) [file 12891_2018_2173_MOESM2_ESM.pdf]
