# Supplementary material for: Agreement among physiotherapists in assessing patient performance of exercises for low-back pain
Source: BMC Musculoskelet Disord. 2018 Jul 27;19:265. doi: 10.1186/s12891-018-2173-9 (PMC6064172; doi:10.1186/s12891-018-2173-9)
Supplement: Supplementary file 3 — Bland and Altman plots of agreement for all physiotherapists for strengthening and stretching exercises. (PDF 163 kb) [file 12891_2018_2173_MOESM3_ESM.pdf]

Appendix 3: Bland and Altman plots of agreement for all physiotherapists

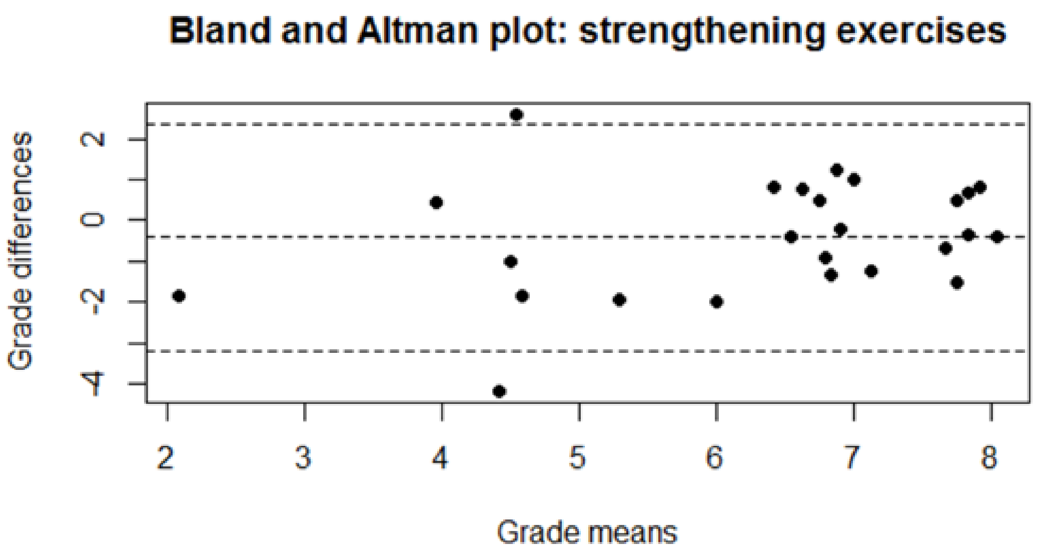

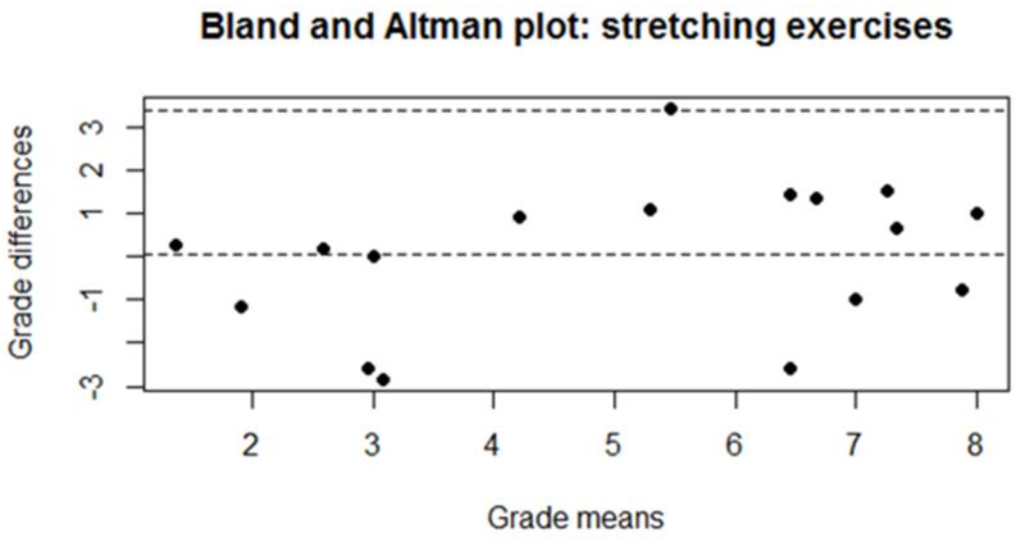

Horizontal dotted line is the mean difference, and upper and lower lines are 95% confidence intervals for limits of agreement.
