# Supplementary material for: Agreement among physiotherapists in assessing patient performance of exercises for low-back pain
Source: BMC Musculoskelet Disord. 2018 Jul 27;19:265. doi: 10.1186/s12891-018-2173-9 (PMC6064172; doi:10.1186/s12891-018-2173-9)
Supplement: Supplementary file 4 — Intra-rater agreement among three rehabilitation centre physiotherapists (face-to-face assessment vs video assessment). (PDF 106 kb) [file 12891_2018_2173_MOESM4_ESM.pdf]

Appendix 4: Intra-rater agreement among three rehabilitation centre physiotherapists (face-to-face assessment vs video assessment)

**Physiotherapist**	***No. of patients assessed***	***Agreement***
**1**	6	0.39 (-0.34-0.72)
**2**	6	0.96 (0.38-1.00)
**3**	4	0.71 (0.1-0.9)

Data are intraclass correlation coefficients (ICCs) and 95% confidence intervals (95% CIs).
